# PgRC2: engineering the compression of sequencing reads

**DOI:** 10.1093/bioinformatics/btaf101

**Published:** 2025-03-04

**Authors:** Tomasz M Kowalski, Szymon Grabowski

**Affiliations:** Institute of Applied Computer Science, Lodz University of Technology, Lodz 90-924, Poland; Institute of Applied Computer Science, Lodz University of Technology, Lodz 90-924, Poland

## Abstract

**Summary:**

The FASTQ format remains at the heart of high-throughput sequencing. Despite advances in specialized FASTQ compressors, they are still imperfect in terms of practical performance tradeoffs. We present a multi-threaded version of Pseudogenome-based Read Compressor (PgRC), an in-memory algorithm for compressing the DNA stream, based on the idea of approximating the shortest common superstring over high-quality reads. Redundancy in the obtained string is efficiently removed by using a compact temporary representation. The current version, v2.0, preserves the compression ratio of the previous one, reducing the compression (resp. decompression) time by a factor of 8–9 (resp. 2–2.5) on a 14-core/28-thread machine.

**Availability and implementation:**

PgRC 2.0 can be downloaded from https://github.com/kowallus/PgRC and https://zenodo.org/records/14882486 (10.5281/zenodo.14882486).

## 1 Introduction

Raw sequencing data in FASTQ format still contribute significantly to the vast volumes of genomic resources. A number of dedicated compression solutions have been proposed in the last decade, mostly focusing on the short reads (typically of length 100 or 150 bp). Most recent algorithms allow removing the redundancy in overlapping reads, to name SCALCE (Hach *et al.* 2012), ORCOM (Grabowski *et al.* 2015), SPRING (Chandak *et al.* 2019), Minicom (Liu *et al.* 2019), FQSqueezer (Deorowicz 2020), PgRC (Kowalski and Grabowski, 2020), and Mstcom (Liu and Li, 2021). SPRING is a reasonable practical option, but if we value compression efficiency even more, the choice may be between PgRC, Mstcom, and FQSqueezer. PgRC 1.1 wins in the compression ratio by up to 15% over SPRING and is rather fast in the decompression, although not in the compression, being a single-threaded program. On the other hand, FQSqueezer is even stronger in compression (by up to around 10%), yet its computational requirements are significant, being many times slower in decompression than PgRC, due to the symmetric nature of the underlying PPM compression scheme. More details about these tools can be found, e.g. in (Kowalski and Grabowski 2020). Mstcom is somewhere in between, offering strong compression and quite acceptable speed.

In this work, we present an improved version of PgRC (v2.0), mostly focused on boosting the compression and the decompression, usually with heuristic techniques. One is parallelization of almost all of its phases, another is a simple DNA preprocessing technique allowing to find LZ-matches much faster. We also make use of the quality stream as a read quality gauge, effectively speeding up the compression phase.

## 2 Materials and methods

PgRC works in several stages (we use this word interchangeably with “phases” throughout the paper), which are now briefly presented; more details can be found in (Kowalski and Grabowski, 2020, Sect. 2). The parallelization, added for most stages, is obtained with aid of the OpenMP library. From the input FASTQ, its DNA stream is extracted (in a single-threaded phase), and an approximation of the shortest common superstring (called later a “pseudogenome” or pg in short) (Kowalski *et al.* 2015) is built over the high-quality reads. The high-quality reads are found during an early step of the pg construction, which is later called “read set division.” In a fast preprocessing, we separate all the reads that contain at least one N symbol (they form a separate read subset) and reads which seem to be of low quality, based on the information taken from the quality stream. To this end, we use a simple heuristics, which checks the quality for a single DNA letter at a fixed position (by default at the distance of 0.12 times the read length from its end, e.g. 12 symbols from the end for 100-symbol-long reads); if its expected error is above some threshold (63%, which corresponds to Phred quality score 2 and below, or equivalently, ASCII codes 35, 34, and 33), the whole read is rejected from the high-quality ones. After this quality score-based filtering, which is novel in the current PgRC version and boosts the read set division phase by a factor of 1.8 on average, in the high-quality subset, we want to keep those reads that produce long enough left and right overlaps with regard to other reads. Once the set of high-quality reads is established, the pseudogenome over them, $PG_{hq}$, is constructed. Finding read overlaps in a multi-threaded implementation required care, since a naive parallel matching may lead to cycles among overlapping reads. In the next stage, the reads that do not participate in $PG_{hq}$ (and thus generally being of lower quality) are mapped onto $PG_{hq}$ with several allowed mismatches (i.e. Hamming errors). More concretely, we allow up to ⌊read_len/M⌋ mismatches per aligned read, where M is 3 by default. This means that for reads of length 100, we allow up to 33 mismatches between the read and the area of $PG_{hq}$ it is aligned to.

Each successfully aligned read is represented with several items of data, sent to multiple streams, and later encoded and compressed using custom techniques. Those data include the offset (position) in $PG_{hq}$ of the aligned read, its number of mismatches, their positions (encoded differentially), the read’s symbols at the mismatching positions, and a flag to tell if the read is mapped forwardly or reverse-complemented. The read alignment stage is now multi-threaded.

From the low-quality reads that have not mapped onto $PG_{hq}$, we create two pseudogenomes, $PG_{lq}$ and $PG_{N}$, where the latter is based on reads containing symbols N. The construction of those pseudogenomes is identical to $PG_{hq}$ and multi-threaded as well.

The high-quality pseudogenome, $PG_{hq}$, contains specific redundancy that would not be removed in the final stage when a general-purpose compression algorithm is applied. Here, we mean reverse-complemented matches of relatively long strings. We find those matches quite efficiently using a hash table and a sparse sampling technique (Grabowski and Bieniecki, 2019). The implementation of the RC-match search itself is currently serial (parallelization might be added in a future version, although the expected overall speedup will be rather small). The mentioned sparse sampling technique is also used in the read alignment phase (here fully multi-threaded), and our lock-free design speeds things up about 17 times on average. The hash table is built using multiple threads, but not all collisions across different threads, which occur during the hash table construction are handled. This means that some of the matches that would be found using a single-threaded implementation may now be missed, but in practice, it is a rare situation (see the Supplementary Material for more information).

What is left is backend compression on the resulting components. Like in the previous PgRC version, we stick to LZMA for the pseudogenomes, but for the remaining components, we use a wider variety of compressors: PPMd, range coder, FSE, and LZMA. There are more changes, though. First (quite trivial) is that we run the LZMA algorithm with two threads, as supported by the algorithm’s API. Various streams are now compressed in parallel. Another change concerns the backend compressor selection. Previously we assigned an appropriate compressor for a particular stream based on an offline, i.e. performed at compressor design time, analysis. Now, we recognize that for some streams, the choice is not obvious, in terms of the expected speed/ratio tradeoff, and allow to assign two or three candidates in such cases. For those streams, selected backend compression options (two or three compressors: FSE, PPMd, and range coder) are tried out at compression time, and the one with higher compression ratio is chosen. For example, FSE is much faster than PPMd and in some cases gives a higher compression ratio, so it pays to sacrifice a little extra time for a chance to squeeze a given stream more tightly.

Another modification is that the stream of mismatches, i.e. the mismatched bases of the mapped read, is now preprocessed with regard to the global order-0 statistics of the mismatches. To give an example, if the most frequent mismatch is the symbol C, then followed by G, A, T, and N, they are initially replaced with their ranks: 0, 1, 2, 3, and 4, respectively, and then the rank of a particular mismatch may be often decreased by 1 due to an exclusion (of the corresponding symbol in the pseudogenome), which effectively reduces the alphabet of mismatch symbols. Following the example, if the symbol from the reference is G, then the rank for the corresponding mismatch C is 0, but the ranks for A, T, and N are only 1, 2, and 3, respectively. The stream of ranks is then sent to a backend coder. This idea is inspired by the encoding from (Xie *et al.* 2022), but simplified; according to our experiments, ignoring the context they use yields more or less the same results.

**Figure 1.**
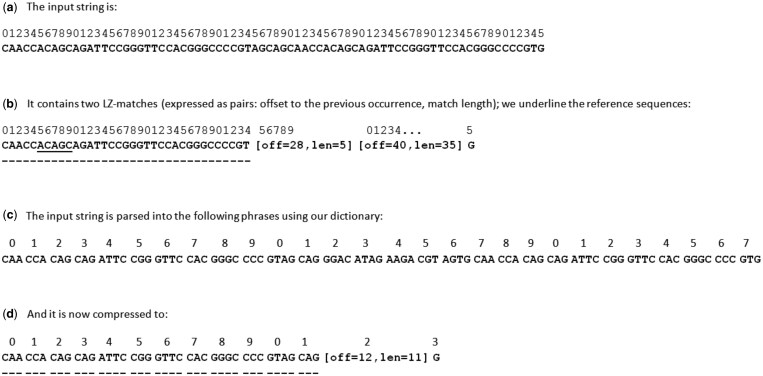
An example of LZ-matches in the natural DNA string (a and b) and the impact of variable-length packing (c) on further LZ-matching (d). The minimum LZ-match length in the original representation is assumed to be 5. Instead of two LZ-matches, of length 5 and 35 symbols, in the compact representation we have only one match spanning over 30 original symbols.

The most significant change is intended to reduce the LZMA compression time by using a more dense input representation of the pseudogenomes. Following the idea from (Bonfield and Mahoney, 2013, p. 8), we pack together a variable number (up to 4) of DNA bases into a byte, in a way that facilitates self-synchronization, which is important not to lose (or shorten by much) too many matches in the further, compression stage (Fig. 1 illustrates). Below, we present details on our packing scheme.

The dictionary of 242 phrases (of length from 1 to 4 bases) is ordered according to their reversals (e.g. TAA is just before ATAA), with some locations in the range 0…255 unused. Due to some technical reasons related to the backend LZMA compressor, and thus slightly improving the compression, we start each group of phrases ending with the new standard symbol (A, C, G, T) at a multiple of 64. Some phrases include the symbols N and %, the latter signaling reverse-complemented matches. The encoder works greedily, always finding the longest prefix of the remaining text corresponding to a phrase in the dictionary. Both the text encoding and decoding using the presented variable-length scheme are rather fast, despite a single-threaded implementation, and achieve around 500 MB/s on our machine, which means that the compression of this stream is often boosted 20 times or more, and the overall speedup (in a multi-threaded PgRC 2.0 experiment) about three-fold. Although transforming the DNA sequence to such packed representation in some cases has a minor negative impact on the final compression (with over 5% loss for this stream on SRR870667_1, which is a clear outlier though), overall we lose <0.1% on average and thus we found this idea posing a viable time-compression tradeoff.

## 3 Results

PgRC 2.0 was tested on a Linux machine with a 14-core Intel Core i9-10940X 3.3 GHz CPU, 128 GB of DDR4-RAM (CL 16, 2666 MHz) and an SSD (ADATA 4 TB M.2 PCIe Legend 960).

The test collection (cf. Sect. 1 in the Supplementary Material) consists of a number of real genome and transcriptome sequencing datasets, used for benchmarking in prior works on FASTQ compression. Dataset sizes are given in GBytes (or Gigabases), where $G=10^{9}$. SPRING, Mstcom and PgRC 2.0 were run with 28 threads and other settings default. PgRC 1.1 is a single-threaded implementation. These three compressors are seemingly the most practical in this application, but there is a recent trend of more versatile tools [e.g. Genozip (Lan *et al.* 2021)], which deal with multiple bioinformatics formats, but due to their streaming regime of work they cannot obtain competitive compression ratios on FASTQ reads. See the Supplementary Material for more results and details on the test methodology.

Tables 1 and 2 present the results in the order non-preserving SE and order-preserving PE modes, respectively (SE and PE_ORD in short). The datasets are mostly the same as those used in our earlier work (Kowalski and Grabowski 2020), while the set of competitors now comprises SPRING, Mstcom and the previous public version of PgRC, v1.1. In both experiments, PgRC 2.0 wins in compression ratio over SPRING in almost all cases (15 out of 16 and 9 out of 9, respectively), but only in 2 cases out of 15 (SE) and 4 cases out of 6 (PE_ORD) over the much slower Mstcom. Please note that Mstcom failed on some datasets and our comparative conclusions only embrace the successful cases. More precisely, we can note that PgRC 2.0 archives are on average by 15.7% smaller than SPRING’s in the SE regime and by 14.0% smaller in PE_ORD, while the average loss to Mstcom is 12.6% and 0.8%, respectively. The compression ratio of PgRC 1.1 is preserved (actually improved by <1%). In compression time (ctime), SPRING is the winner, being by 6% and 11% faster than PgRC 2.0, which in turn beats Mstcom 10–11 times. In decompression (dtime), PgRC 2.0 takes the lead, being 1.5 (resp. 1.4) times faster than SPRING in the SE (resp. PE_ORD) mode, while Mstcom is 8 (resp. 17) times slower. We also stress that the single-threaded PgRC 1.1 is beaten by v2.0 by almost an order of magnitude in ctime and at least twice in dtime. Importantly, the pseudogenome compression time for PgRC 2.0 even with 1 thread is over 15 times shorter than the corresponding time for PgRC 1.1. This spectacular improvement was mainly possible due to the variable-length encoding phase, which is almost free in the processing time, and not only shortens the input for the LZMA, but also speeds up match searching over it, thanks to its more “dense” representation. All the averages presented in this section are geometric means.

**Table 1.** Compression and time in the order non-preserving regime on SE datasets.^a^

Dataset	Size	SPRING			Mstcom			PgRC 1.1			PgRC 2.0		
	(Gbs)	Ratio	ctime	dtime	Ratio	ctime	dtime	Ratio	ctime	dtime	Ratio	ctime	dtime
ERR3239279_1	63.03	0.1872	**1430.7**	100.8	–	–	–	0.1574	11577.0	127.3	**0.1548**	1522.1	**81.2**
ERP001775_1	44.39	0.2297	**1094.6**	82.7	0.2194	19114.0	543.1	0.2190	10628.0	111.2	**0.2174**	1365.5	**77.7**
ERR174310_1	20.97	0.4092	**630.0**	**45.9**	**0.3671**	7895.0	393.0	0.4509	9639.0	92.5	0.4484	1020.5	55.5
SRR870667_1	7.48	1.2621	**326.5**	**29.8**	**0.5262**	3434.6	192.7	0.7655	8901.0	56.6	0.8556	680.9	32.4
SRR445724	5.09	0.4356	194.6	13.4	**0.2917**	1690.2	77.1	0.3401	1736.6	20.2	0.3403	**151.8**	**8.7**
SRR445726	4.92	0.3905	175.3	11.2	**0.2656**	1561.3	68.4	0.3090	1515.4	18.3	0.3082	**136.8**	**7.9**
SRR490961	4.91	0.2203	108.1	9.6	**0.1591**	1190.8	44.9	0.1794	840.2	12.3	0.1764	**90.7**	**5.4**
SRR1294116	4.64	0.2770	**81.3**	9.6	**0.2144**	882.0	53.9	0.2333	963.5	12.8	0.2301	96.8	**7.1**
ERR532393_1	3.58	0.4330	**70.3**	8.8	**0.3172**	1439.8	59.5	0.3711	1147.3	13.9	0.3745	98.1	**8.3**
SRR065390_1	3.38	0.2284	**66.3**	7.4	0.1902	837.0	38.2	**0.1858**	526.1	8.1	0.1914	66.5	**4.0**
SRR490976	3.33	0.3927	118.8	9.3	**0.2876**	1165.2	48.7	0.3316	1048.8	12.7	0.3228	**92.0**	**5.5**
SRR445718	3.29	0.3409	79.9	7.9	**0.2461**	880.6	43.0	0.2795	864.2	11.1	0.2744	**79.0**	**5.0**
SRR689233_1	1.48	0.1949	**27.4**	4.3	**0.1460**	278.5	13.0	0.1687	214.0	3.6	0.1663	28.0	**1.7**
SRR635193_1	1.47	0.2687	35.9	4.5	**0.2190**	265.4	18.5	0.2558	277.1	4.2	0.2481	**32.2**	**2.5**
MiSeq_1	0.87	0.1258	12.1	2.0	0.0986	141.2	5.6	0.0937	76.7	1.5	**0.0914**	**11.3**	**0.7**
SRR554369_1	0.17	0.2416	3.2	0.6	**0.2303**	23.9	2.0	0.2393	20.6	0.5	0.2408	**3.0**	**0.3**

a Compression ratios are in bits per base (bpb). “ctime” (resp. “dtime”) stands for compression (resp. decompression) time, and is expressed in seconds. The best results in the given row are bolded.

**Table 2.** Compression and time in the order-preserving regime on PE datasets.^a^

Dataset	size	SPRING			Mstcom			PgRC 1.1			PgRC 2.0		
	(Gbs)	Ratio	ctime	dtime	Ratio	ctime	dtime	Ratio	ctime	dtime	Ratio	ctime	dtime
ERR3239279	126.06	0.2460	**2920.8**	233.6	–	–	–	0.1762	20599.0	390.5	**0.1741**	2980.5	**179.1**
ERP001775	88.78	0.3848	**1990.2**	194.0	–	–	–	0.3399	19599.0	467.1	**0.3395**	2830.0	**166.5**
ERR174310	41.93	0.4643	**1042.1**	96.3	0.4367	17687.0	1930.2	0.4388	13314.0	244.3	**0.4361**	1780.5	**93.1**
ERR532393	7.15	0.6205	**148.3**	21.4	**0.4818**	3152.6	259.9	0.5235	2117.8	47.5	0.5210	190.4	**15.7**
SRR065390	6.76	0.3951	134.6	15.8	0.3384	1861.1	214.3	**0.3190**	965.6	33.0	0.3203	**116.0**	**12.6**
SRR689233	2.95	0.3693	**58.2**	8.0	**0.3250**	633.0	89.9	0.3538	433.4	14.5	0.3497	59.1	**5.2**
SRR635193	2.94	0.5653	72.5	9.6	0.5382	546.1	186.7	0.5382	553.2	20.8	**0.5370**	**72.1**	**8.3**
MiSeq	1.73	0.2753	**24.8**	4.2	–	–	–	0.2174	204.9	5.8	**0.2112**	26.9	**2.2**
SRR554369	0.33	0.3237	5.7	1.2	0.3167	49.8	7.5	0.3086	36.3	1.4	**0.3031**	**4.8**	**0.6**

a Compression ratios are in bits per base (bpb). “ctime” (resp. “dtime”) stands for compression (resp. decompression) time, and is expressed in seconds. The best results in the given row are bolded.

To sum up, we can say that SPRING is roughly comparable in speed (ctime and dtime) to PgRC 2.0, but the compression difference is ∼10%–15%. Mstcom offers a boost in compression ratio, by up to around 15%, but is an order of magnitude slower in compression and decompression, and is not very stable. PgRC 2.0, using 28 threads on a 14-core/28-thread machine, is faster than PgRC 1.1 by a factor of ∼8–9 in compression and 2–2.5 in decompression, respectively, while preserving, or even slightly improving, its compression ratio.

## 4 Conclusions

PgRC 2.0 is a multi-threaded tool for compressing the DNA stream, improving the compression time of its previous version (v1.1) by a factor of ∼8–9; the parallelization itself gives a boost of factor ∼5. One of the key ideas behind its success was to pack the DNA stream (from the pseudogenomes) into bytes, using a variable-length encoding, which speeds up the later LZMA compression even more than could be expected from the more than threefold reduction in the length, thanks to making the data more dense for the LZ77-like match finding.

We note that PgRC is still work in progress, and some other engineering (and possibly algorithmic) improvements are possible. In particular, we are going to release a full-fledged FASTQ compressor, handling all data streams, including the quality scores and read headers.

## Supplementary Material

btaf101_Supplementary_Data

## Data Availability

The data underlying this article are available in two public repositories, with their URLs given in Sec. 1 of the supplementary material.
