# Supplementary material for: PgRC2: engineering the compression of sequencing reads
Source: Bioinformatics. 2025 Mar 4;41(3):btaf101. doi: 10.1093/bioinformatics/btaf101 (PMC11908645; doi:10.1093/bioinformatics/btaf101)
Supplement: btaf101_Supplementary_Data [file btaf101_supplementary_data.pdf]

Supplementary Material to: “PgRC2: Engineering the Compression of Sequencing Reads”

Tomasz M. Kowalski and Szymon Grabowski

February 2025

1 Dataset URLs

Most of the dataset descriptions are taken from <https://www.ebi.ac.uk>.

ERP001775 (H. sapiens)

https://ftp.sra.ebi.ac.uk/vol1/fastq/ERR174/ERR174324/ERR174324_1.fastq.gz

https://ftp.sra.ebi.ac.uk/vol1/fastq/ERR174/ERR174325/ERR174325_1.fastq.gz

https://ftp.sra.ebi.ac.uk/vol1/fastq/ERR174/ERR174324/ERR174324_2.fastq.gz

https://ftp.sra.ebi.ac.uk/vol1/fastq/ERR174/ERR174325/ERR174325_2.fastq.gz

The first two files were concatenated and the last two files were concatenated to obtain a 28x coverage paired-end dataset.

ERR174310 (H. sapiens)

https://ftp.sra.ebi.ac.uk/vol1/fastq/ERR174/ERR174310/ERR174310_1.fastq.gz

https://ftp.sra.ebi.ac.uk/vol1/fastq/ERR174/ERR174310/ERR174310_2.fastq.gz

ERR194146 (H. sapiens)

https://ftp.sra.ebi.ac.uk/vol1/fastq/ERR194/ERR194146/ERR194146_1.fastq.gz

https://ftp.sra.ebi.ac.uk/vol1/fastq/ERR194/ERR194146/ERR194146_2.fastq.gz

ERR3239279 (H. sapiens, 1000 Genomes Project Phase 3)

https://ftp.sra.ebi.ac.uk/vol1/fastq/ERR323/009/ERR3239279/ERR3239279_1.fastq.gz

https://ftp.sra.ebi.ac.uk/vol1/fastq/ERR323/009/ERR3239279/ERR3239279_2.fastq.gz

ERR532393 (Population Genomics of metagenome sequence)

https://ftp.sra.ebi.ac.uk/vol1/fastq/ERR532/ERR532393/ERR532393_1.fastq.gz

https://ftp.sra.ebi.ac.uk/vol1/fastq/ERR532/ERR532393/ERR532393_2.fastq.gz

MiSeq (E. coli)

ftp://webdata:webdata@usssd-ftp.illumina.com/Data/SequencingRuns/MG1655/MiSeq_Ecoli_MG1655_110721_PF_R1.fastq.gz

ftp://webdata:webdata@usssd-ftp.illumina.com/Data/SequencingRuns/MG1655/MiSeq_Ecoli_MG1655_110721_PF_R2.fastq.gz

SRR065390 (Illumina Genome Analyzer II paired end sequencing)

https://ftp.sra.ebi.ac.uk/vol1/fastq/SRR065/SRR065390/SRR065390_1.fastq.gz

https://ftp.sra.ebi.ac.uk/vol1/fastq/SRR065/SRR065390/SRR065390_2.fastq.gz

SRR1294116 (Illumina HiSeq 2000 sequencing; GSM1395310: human ES cell line H9 (ucla_h9); Homo sapiens; RNA-Seq)

<https://ftp.sra.ebi.ac.uk/vol1/fastq/SRR129/006/SRR1294116/SRR1294116.fastq.gz>

SRR445718 (Illumina HiSeq 2000 sequencing; GSM896803: Oocyte #1-C1; Homo sapiens; RNA-Seq)

<https://ftp.sra.ebi.ac.uk/vol1/fastq/SRR445/SRR445718/SRR445718.fastq.gz>

SRR445724 (Illumina HiSeq 2000 sequencing; GSM896809: 2-cell #1-C1; Homo sapiens; RNA-Seq)
<https://ftp.sra.ebi.ac.uk/vol1/fastq/SRR445/SRR445724/SRR445724.fastq.gz>

SRR445726 (Illumina HiSeq 2000 sequencing; GSM896811: 2-cell #2-C1; Homo sapiens; RNA-Seq)
<https://ftp.sra.ebi.ac.uk/vol1/fastq/SRR445/SRR445726/SRR445726.fastq.gz>

SRR490961 (Illumina HiSeq 2000 sequencing; GSM922146: 4-cell embryo#1 -Cell#1; Homo sapiens; RNA-Seq)
<https://ftp.sra.ebi.ac.uk/vol1/fastq/SRR490/SRR490961/SRR490961.fastq.gz>

SRR490976 (Illumina HiSeq 2000 sequencing; GSM922161: 8-cell embryo#1 -Cell#4; Homo sapiens; RNA-Seq)
<https://ftp.sra.ebi.ac.uk/vol1/fastq/SRR490/SRR490976/SRR490976.fastq.gz>

SRR554369 (P. aeruginosa)
https://ftp.sra.ebi.ac.uk/vol1/fastq/SRR554/SRR554369/SRR554369_1.fastq.gz
https://ftp.sra.ebi.ac.uk/vol1/fastq/SRR554/SRR554369/SRR554369_2.fastq.gz

SRR635193 (Illumina Genome Analyzer IIx paired end sequencing; pooled amnion from 5 term birth placentas)
https://ftp.sra.ebi.ac.uk/vol1/fastq/SRR635/SRR635193/SRR635193_1.fastq.gz
https://ftp.sra.ebi.ac.uk/vol1/fastq/SRR635/SRR635193/SRR635193_2.fastq.gz

SRR689233 (Illumina HiSeq 2000 paired end sequencing; GSM1080195: mouse oocyte 1; Mus musculus; RNA-Seq)
https://ftp.sra.ebi.ac.uk/vol1/fastq/SRR689/SRR689233/SRR689233_1.fastq.gz
https://ftp.sra.ebi.ac.uk/vol1/fastq/SRR689/SRR689233/SRR689233_2.fastq.gz

SRR870667_1 (T. cacao)
https://ftp.sra.ebi.ac.uk/vol1/fastq/SRR870/SRR870667/SRR870667_1.fastq.gz

2 Tested programs

The following programs, with the set parameters (e.g., for number of threads set to 28), were used in our experiments, with their results presented either in the main paper or in Section 3 of the Supplementary data.

SPRING

(version 1.1.1 from 2023-May-28, <https://github.com/shubhamchandak94/Spring/releases/tag/v1.1.1>):

SE, compression of DNA stream:

```
spring -c --no-ids --no-quality -i <fastq-in-file> -o <archive-file> -t 28 -r
```

SE_ORD, compression of DNA stream:

```
spring -c --no-ids --no-quality -i <fastq-in-file> -o <archive-file> -t 28
```

PE, compression of DNA stream:

```
spring -c --no-ids --no-quality -i <fastq-in-file> <fastq-pair-in-file> -o <archive-file> -t 28 -r
```

PE_ORD, compression of DNA stream:

```
spring -c --no-ids --no-quality -i <fastq-in-file> <fastq-pair-in-file> -o <archive-file> -t 28
```

SE and SE_ORD, decompression of DNA stream:

```
spring -d -i <archive-file> -o <fastq-out-file> -t 28
```

PE and PE_ORD, decompression of DNA stream:

```
spring -d -i <archive-file> -o <fastq-out-file> <fastq-pair-out-file> -t 28
```

Mstcom

(version from 2021-Apr-12, <https://github.com/yuansliu/mstcom>):

SE, compression of DNA stream:

```
mstcom e -i <fastq-in-file> -o <archive-file> -t 28
```

SE_ORD, compression of DNA stream:

```
mstcom e -i <fastq-in-file> -o <archive-file> -t 28 -p
```

PE, compression of DNA stream:

```
mstcom e -i <fastq-in-file> -f <fastq-pair-in-file> -o <archive-file> -t 28
```

PE_ORD, compression of DNA stream:

```
mstcom e -i <fastq-in-file> -f <fastq-pair-in-file> -o <archive-file> -t 28 -p
```

decompression of DNA stream:

```
mstcom d -i <archive-file> -o <out-path-prefix> -t 28
```

PgRC v1.1

(version 1.1 from 2020-Apr-23, <https://github.com/kowallus/PgRC/releases/tag/v1.1>):

SE, compression of DNA stream:

```
PgRC -i <fastq-in-file> -o <archive-file>
```

SE_ORD, compression of DNA stream:

```
PgRC -o -i <fastq-in-file> <archive-file>
```

PE, compression of DNA stream:

```
PgRC -i <fastq-in-file> <fastq-pair-in-file> <archive-file>
```

PE_ORD, compression of DNA stream:

```
PgRC -o -i <fastq-in-file> <fastq-pair-in-file> <archive-file>
```

decompression of DNA stream:

```
PgRC -d <archive-file>
```

PgRC v2.0

(version 2.0 from 2024-Sep-26, <https://github.com/kowallus/PgRC/releases/tag/v2.0>, <https://zenodo.org/records/14882486>):

SE, compression of DNA stream:

```
PgRC -t 28 -i <fastq-in-file> -o <archive-file>
```

SE_ORD, compression of DNA stream:

```
PgRC -o -t 28 -i <fastq-in-file> <archive-file>
```

PE, compression of DNA stream:

```
PgRC -t 28 -i <fastq-in-file> <fastq-pair-in-file> <archive-file>
```

PE_ORD, compression of DNA stream:

```
PgRC -o -t 28 -i <fastq-in-file> <fastq-pair-in-file> <archive-file>
```

decompression of DNA stream:

```
PgRC -d -t 28 <archive-file>
```

Genie

(version from 2023-Sep-9, <https://github.com/MueFab/genie>):

SE, compression of DNA stream (with transcoding from FASTQ into MPEG-G records)

```
genie transcode-fastq -i <fastq-in-file> -o <mpegrec-file> -t 28
```

```
genie run --read-ids none --qv none -i <mpegrec-file> -o <archive-file> -t 28
```

SE, decompression of DNA stream (with transcoding from MPEG-G records back to FASTQ):

```
genie run -i <archive-file> -o <mpegrec-file> -t 28
```

```
genie transcode-fastq -i <mpegrec-file> -o <fastq-out-file> -t 28
```

Genozip

(version 15.0.68 from 2024-Oct-13, <https://github.com/divonlan/genozip/releases/tag/genozip-15.0.68>):

FASTQ compression:

```
genozip <fastq-in-file> --stats -o <archive-file> --threads 28
```

FASTQ compression the best mode:

```
genozip <fastq-in-file> --stats --best -o <archive-file> --threads 28
```

FASTQ decompression:

```
genounzip <archive-file> -o <fastq-out-file> --threads 28
```

Note that to check the size of the compressed DNA stream we need to use the `--stats` option which displays the internal structure of a genozip file and the associated compression statistics.

Jarvis 3

(version 3.7 from 2024-Oct-28, <https://github.com/cobilab/jarvis3/releases/tag/v3.7>):

FASTQ compression:

```
JARVIS3.sh --level 7 --fastq --input <fastq-in-file> --threads 28
```

FASTQ decompression:

```
JARVIS3.sh --decompress --fastq --input <archive-file> --threads 28
```

Note that 7 is this tool's default compression level and the archive file name is `<fastq-in-file>.tar`. Jarvis 3, similarly to genozip with the `--stats` switch, displays the sizes of all compressed FASTQ streams.

dataset	size<br>[Gbs]	SPRING					Mstcom					PgRC 1.1					PgRC 2.0				
		ratio	ctime	dtime	cmem	dmem	ratio	ctime	dtime	cmem	dmem	ratio	ctime	dtime	cmem	dmem	ratio	ctime	dtime	cmem	dmem
ERR3239279_1	63.03	0.1872	**1430.7**	100.8	**26.07**	**8.39**	—	—	—	—	—	0.1574	11577.0	127.3	40.76	17.55	**0.1548**	1522.1	**81.2**	40.75	17.96
ERP001775_1	44.39	0.2297	**1094.6**	82.7	**23.82**	**5.91**	0.2194	19114.0	543.1	127.70	66.76	0.2190	10628.0	111.2	38.20	16.39	**0.2174**	1365.5	**77.7**	37.94	16.52
ERR174310_1	20.97	0.4092	**630.0**	**45.9**	**11.57**	**6.31**	**0.3671**	7895.0	393.0	74.09	32.79	0.4509	9639.0	92.5	21.36	12.24	0.4484	1020.5	55.5	20.98	12.80
SRR870667_1	7.48	1.2621	**326.5**	**29.8**	**9.59**	**6.46**	**0.5262**	3434.6	192.7	45.53	16.11	0.7655	8901.0	56.6	19.45	12.58	0.8556	680.9	32.4	12.90	10.50
SRR445724	5.09	0.4356	194.6	13.4	5.85	4.87	**0.2917**	1690.2	77.1	34.87	5.29	0.3401	1736.6	20.2	7.10	2.57	0.3403	**151.8**	**8.7**	**3.68**	**2.14**
SRR445726	4.92	0.3905	175.3	11.2	5.49	4.70	**0.2656**	1561.3	68.4	33.19	4.87	0.3090	1515.4	18.3	6.39	2.24	0.3082	**136.8**	**7.9**	**3.36**	**1.89**
SRR490961	4.91	0.2203	108.1	9.6	4.41	2.96	**0.1591**	1190.8	44.9	26.22	4.52	0.1794	840.2	12.3	2.79	**1.09**	0.1764	**90.7**	**5.4**	**2.52**	1.23
SRR1294116	4.64	0.2770	**81.3**	9.6	4.17	3.16	**0.2144**	882.0	53.9	24.58	4.13	0.2333	963.5	12.8	3.83	**1.26**	0.2301	96.8	**7.1**	**2.30**	1.31
ERR532393_1	3.58	0.4330	**70.3**	8.8	4.37	3.68	**0.3172**	1439.8	59.5	22.35	4.89	0.3711	1147.3	13.9	5.30	**1.27**	0.3745	98.1	**8.3**	**2.35**	1.38
SRR065390_1	3.38	0.2284	**66.3**	7.4	3.92	2.05	0.1902	837.0	38.2	23.90	4.90	**0.1858**	526.1	8.1	1.89	**0.76**	0.1914	66.5	**4.0**	**1.77**	1.00
SRR490976	3.33	0.3927	118.8	9.3	4.78	3.99	**0.2876**	1165.2	48.7	27.23	3.77	0.3316	1048.8	12.7	4.38	1.59	0.3228	**92.0**	**5.5**	**2.42**	**1.34**
SRR445718	3.29	0.3409	79.9	7.9	4.09	3.03	**0.2461**	880.6	43.0	24.33	3.77	0.2795	864.2	11.1	3.36	1.15	0.2744	**79.0**	**5.0**	**2.10**	**1.12**
SRR689233_1	1.48	0.1949	**27.4**	4.3	3.60	1.61	**0.1460**	278.5	13.0	11.50	1.89	0.1687	214.0	3.6	**0.76**	**0.31**	0.1663	28.0	**1.7**	0.81	0.43
SRR635193_1	1.47	0.2687	35.9	4.5	2.22	1.41	**0.2190**	265.4	18.5	12.45	1.48	0.2558	277.1	4.2	1.66	**0.49**	0.2481	**32.2**	**2.5**	**1.01**	0.63
MiSeq_1	0.87	0.1258	12.1	2.0	3.07	1.40	0.0986	141.2	5.6	7.54	1.15	0.0937	76.7	1.5	**0.36**	**0.10**	**0.0914**	**11.3**	**0.7**	0.39	0.20
SRR554369_1	0.17	0.2416	3.2	0.6	1.38	0.32	**0.2303**	23.9	2.0	3.75	0.26	0.2393	20.6	0.5	**0.21**	**0.05**	0.2408	**3.0**	**0.3**	0.25	0.17
ERR3239279	126.06	0.1964	2997.1	224.1	**50.86**	**11.85**	—	—	—	—	—	0.1542	17850.0	315.0	54.20	25.44	**0.1516**	**2461.3**	**146.6**	54.21	25.18
ERP001775	88.78	0.2312	**1993.4**	177.1	46.54	**7.33**	—	—	—	—	—	0.2026	15532.0	268.5	46.79	24.76	**0.2004**	2071.3	**120.1**	**46.28**	24.62
ERR174310	41.93	0.3183	**1044.7**	90.0	**22.47**	**6.80**	**0.2948**	17633.0	1651.6	126.21	124.59	0.3062	11476.0	166.0	39.19	17.94	0.3042	1401.9	**81.8**	38.93	18.37
ERR532393	7.15	0.5025	**148.8**	19.1	6.57	5.54	**0.3552**	3395.4	225.1	35.94	20.16	0.4106	2094.3	40.5	9.03	**2.56**	0.4098	190.5	**15.1**	**4.97**	2.84
SRR065390	6.76	0.2696	137.4	15.5	6.07	3.25	0.2127	1846.3	180.4	34.88	19.33	**0.2050**	935.2	22.2	3.40	**1.84**	0.2075	**111.5**	**7.7**	**3.31**	1.96
SRR689233	2.95	0.2414	59.3	8.3	4.31	2.75	**0.1966**	629.6	72.9	18.85	7.48	0.2388	416.9	12.1	**1.47**	**0.96**	0.2353	**57.8**	**3.9**	1.59	1.00
SRR635193	2.94	0.3413	74.3	9.5	2.93	2.27	**0.3104**	534.9	158.3	20.46	7.62	0.3697	519.1	15.9	2.67	**1.33**	0.3656	**70.0**	**5.1**	**1.93**	1.44
MiSeq	1.73	0.2009	**25.6**	4.0	3.35	2.51	—	—	—	—	—	0.1489	200.9	4.8	**0.75**	**0.32**	**0.1444**	26.4	**1.6**	0.79	0.37
SRR554369	0.33	0.2236	5.5	1.0	1.46	0.57	0.2177	49.5	6.2	8.00	0.95	0.2165	35.4	1.1	**0.30**	**0.10**	**0.2115**	**4.5**	**0.5**	0.33	0.19

Table 1: Compression, time and memory usage in the order non-preserving regime on SE datasets (top 16 rows) and PE datasets (bottom 9 rows). The best results in the given row are bolded. Compression ratios are in bits per base (bpb). “ctime” (resp. “dtime”) stands for compression (resp. decompression) time, and is expressed in seconds. “cmem” (resp. “dmem”) stands for peak compression (resp. decompression) memory usage and is expressed in gigabytes ($=10^9$ bytes).

Mstcom failed to compress some datasets, denoted with “—”, due to excessive memory usage except for MiSeq which was interrupted by segmentation fault.

dataset	size<br>[Gbs]	SPRING					Mstcom					PgRC 1.1					PgRC 2.0				
		ratio	ctime	dtime	cmem	dmem	ratio	ctime	dtime	cmem	dmem	ratio	ctime	dtime	cmem	dmem	ratio	ctime	dtime	cmem	dmem
ERR3239279_1	63.03	0.2954	**1440.3**	112.0	**26.07**	**5.07**	—	—	—	—	—	0.2083	13991.0	153.2	44.12	20.49	**0.2062**	2066.8	**82.8**	44.02	20.85
ERP001775_1	44.39	0.5293	**1109.0**	**100.3**	**23.82**	**4.68**	0.5097	18991.0	1149.0	127.81	71.54	0.5052	13919.0	193.3	41.72	19.46	**0.5013**	2201.9	125.8	41.31	19.77
ERR174310_1	20.97	0.6967	**605.5**	**54.0**	**11.57**	**4.76**	**0.6425**	7844.0	718.5	74.33	34.52	0.7275	11004.0	136.2	24.85	13.69	0.7144	1398.9	64.2	22.61	14.15
SRR870667_1	7.48	1.4344	**326.6**	**31.6**	**8.06**	**4.31**	**0.7755**	3465.8	288.9	45.68	12.90	1.0305	8955.0	69.7	20.04	12.79	1.1143	699.0	33.3	13.45	11.07
SRR445724	5.09	0.6637	198.7	13.8	4.12	**2.23**	**0.5291**	1697.3	137.8	34.52	8.40	0.5937	1793.4	28.1	7.51	2.72	0.5927	**166.4**	**12.2**	**3.95**	2.30
SRR445726	4.92	0.6191	179.8	13.2	4.12	2.12	**0.5007**	1570.0	127.3	32.72	8.08	0.5601	1568.5	25.6	6.79	2.39	0.5577	**150.9**	**11.3**	**3.62**	**2.02**
SRR490961	4.91	0.4607	112.5	11.2	4.13	1.74	**0.3985**	1197.4	101.0	28.51	7.94	0.4286	896.7	19.9	3.36	1.24	0.4255	**104.2**	**10.9**	**2.82**	**1.17**
SRR1294116	4.64	0.5137	**84.1**	11.4	4.09	1.83	**0.4449**	890.2	108.2	24.35	7.44	0.4740	1014.4	19.3	4.19	1.40	0.4726	110.1	**10.2**	**2.52**	**1.32**
ERR532393_1	3.58	0.6663	**71.6**	10.0	4.07	2.01	**0.5587**	1402.4	98.1	22.53	5.87	0.6155	1177.0	19.7	5.59	**1.38**	0.6168	106.6	**9.1**	**2.53**	1.47
SRR065390_1	3.38	0.4784	**67.4**	**7.9**	3.93	1.73	0.4334	835.4	76.4	23.91	5.51	**0.4258**	554.6	13.6	2.46	**0.84**	0.4305	74.2	8.0	**1.98**	0.95
SRR490976	3.33	0.6066	118.1	9.2	4.05	2.32	**0.5210**	1172.0	84.8	27.51	5.46	0.5777	1088.2	17.8	4.64	1.68	0.5656	**102.7**	**7.5**	**2.59**	**1.44**
SRR445718	3.29	0.5754	**82.4**	8.7	4.08	1.80	**0.4806**	884.5	81.6	24.35	5.38	0.5282	903.6	16.1	3.62	1.25	0.5225	87.3	**7.6**	**2.29**	**1.14**
SRR689233_1	1.48	0.4505	**27.1**	3.9	3.60	1.64	**0.3960**	281.9	31.8	12.12	2.65	0.4289	233.0	5.6	1.12	**0.36**	0.4258	32.4	**3.4**	**0.84**	0.42
SRR635193_1	1.47	0.6987	**37.2**	5.0	2.22	1.31	**0.6206**	269.9	49.7	12.57	3.06	0.6799	313.0	7.6	1.87	**0.57**	0.6732	42.3	**4.7**	**1.17**	0.63
MiSeq_1	0.87	0.2724	**11.6**	1.9	3.07	1.33	0.2428	141.1	11.6	6.82	1.21	0.2400	81.3	2.2	0.45	**0.11**	**0.2352**	12.8	**1.4**	**0.41**	0.24
SRR554369_1	0.17	0.4437	**3.4**	0.7	1.38	0.32	**0.4299**	23.6	3.6	3.50	0.28	0.4362	21.6	0.7	**0.22**	**0.06**	0.4364	3.4	**0.4**	0.26	0.18
ERR3239279	126.06	0.2460	**2920.8**	233.6	**50.86**	**8.52**	—	—	—	—	—	0.1762	20599.0	390.5	54.20	27.53	**0.1741**	2980.5	**179.1**	54.20	27.89
ERP001775	88.78	0.3848	**1990.2**	194.0	**46.54**	**6.05**	—	—	—	—	—	0.3399	19599.0	467.1	50.31	26.07	**0.3395**	2830.0	**166.5**	49.87	26.45
ERR174310	41.93	0.4643	**1042.1**	96.3	**22.46**	**5.97**	0.4367	17687.0	1930.2	126.20	124.59	0.4388	13314.0	244.3	40.84	19.18	**0.4361**	1780.5	**93.1**	41.50	20.43
ERR532393	7.15	0.6205	**148.3**	21.4	**5.06**	3.70	**0.4818**	3152.6	259.9	36.03	20.15	0.5235	2117.8	47.5	9.33	**2.43**	0.5210	190.4	**15.7**	5.24	2.74
SRR065390	6.76	0.3951	134.6	15.8	6.07	3.12	0.3384	1861.1	214.3	35.13	19.33	**0.3190**	965.6	33.0	**3.61**	**1.28**	0.3203	**116.0**	**12.6**	3.70	1.81
SRR689233	2.95	0.3693	**58.2**	8.0	4.31	2.92	**0.3250**	633.0	89.9	19.84	7.48	0.3538	433.4	14.5	1.72	**0.65**	0.3497	59.1	**5.2**	**1.67**	0.86
SRR635193	2.94	0.5653	72.5	9.6	2.75	2.18	0.5382	546.1	186.7	19.13	7.62	0.5382	553.2	20.8	2.89	**0.94**	**0.5370**	**72.1**	**8.3**	**2.24**	1.34
MiSeq	1.73	0.2753	**24.8**	4.2	3.34	2.51	—	—	—	—	—	0.2174	204.9	5.8	**0.75**	**0.21**	**0.2112**	26.9	**2.2**	0.80	0.39
SRR554369	0.33	0.3237	5.7	1.2	1.46	0.57	0.3167	49.8	7.5	6.11	0.95	0.3086	36.3	1.4	**0.31**	**0.08**	**0.3031**	**4.8**	**0.6**	0.33	0.20

Table 2: Compression, time and memory usage in the order preserving regime on SE datasets (top 16 rows) and PE datasets (bottom 9 rows).

The best results in the given row are bolded. Compression ratios are in bits per base (bpb). “ctime” (resp. “dtime”) stands for compression (resp. decompression) time, and is expressed in seconds. “cmem” (resp. “dmem”) stands for peak compression (resp. decompression) memory usage and is expressed in gigabytes (=10⁹ bytes).

Mstcom failed to compress some datasets, denoted with “—”, due to excessive memory usage except for MiSeq which was interrupted by segmentation fault.

dataset	size<br>[Gbs]	Genozip default					Genozip best					Genie					Jarvis 3				
		ratio	ctime	dtime	cmem	dmem	ratio	ctime	dtime	cmem	dmem	ratio	ctime	dtime	cmem	dmem	ratio	ctime	dtime	cmem	dmem
ERR3239279_1	63.03	0.4225	2319.6	97.3	6.04	1.89	0.4088	3313.0	101.4	92.05	28.84	0.1979	2604.0	136.4	26.29	4.99	0.5812	5132.0	5447.0	0.34	0.34
ERP001775_1	44.39	1.8577	614.8	249.3	4.21	3.55	1.7803	757.2	243.7	67.09	55.55	0.2409	2277.3	126.7	26.56	4.52	1.8208	7810.0	8250.0	0.34	0.34
ERR174310_1	20.97	1.8437	333.5	136.9	3.96	3.01	1.8028	407.4	131.7	67.60	52.09	0.4335	1128.8	63.6	14.24	4.47	1.8215	3713.0	3881.0	0.34	0.34
SRR870667_1	7.48	1.7233	117.1	43.2	4.26	3.34	1.6084	185.9	56.4	59.07	45.91	1.3187	500.0	32.0	9.92	4.68	1.5939	1288.5	1358.9	0.34	0.34
SRR445724	5.09	1.4680	75.2	27.5	4.41	3.40	1.0705	98.3	32.4	50.12	33.42	0.4894	373.5	16.2	5.57	4.53	1.0954	879.9	937.0	0.34	0.34
SRR445726	4.92	1.4002	71.9	26.2	4.35	3.35	1.0285	98.0	32.5	50.07	32.26	0.4391	378.5	15.8	5.31	4.56	1.0643	845.7	886.6	0.34	0.34
SRR490961	4.91	1.5730	71.8	25.8	4.73	3.60	1.1111	98.8	32.0	58.21	32.24	0.2400	312.7	14.3	5.23	4.59	1.0393	845.0	883.9	0.34	0.34
SRR1294116	4.64	1.7020	67.1	25.4	4.22	3.36	1.2780	90.6	31.2	46.42	30.33	0.2829	169.9	13.7	5.18	4.59	1.1385	796.2	844.0	0.34	0.34
ERR532393_1	3.58	1.9946	60.7	23.7	4.31	3.36	1.7576	91.6	31.1	35.62	24.21	0.4451	9.1	2.1	1.50	0.59	1.3951	652.2	665.2	0.34	0.34
SRR065390_1	3.38	1.8470	23.8	8.5	3.67	2.29	1.6738	98.8	21.6	41.36	21.83	0.2407	131.8	10.2	4.87	4.53	1.5053	531.1	557.6	0.34	0.34
SRR490976	3.33	1.4527	50.4	18.4	4.37	3.53	1.0819	82.2	25.9	34.15	22.04	0.4550	65.4	3.5	3.82	3.88	1.0667	578.5	599.2	0.34	0.34
SRR445718	3.29	1.6350	51.8	18.7	4.48	3.30	1.1145	83.0	25.2	41.83	21.69	0.3706	197.9	10.1	4.81	4.59	1.0684	571.7	599.9	0.34	0.34
SRR689233_1	1.48	1.6207	23.1	8.6	4.04	3.11	1.0100	62.5	19.5	16.24	9.69	0.2003	62.9	5.0	3.95	4.47	0.9837	245.4	261.4	0.34	0.34
SRR635193_1	1.47	1.6192	21.0	7.8	3.72	2.85	1.2472	54.2	17.5	15.81	9.62	0.2770	82.8	7.2	3.84	4.21	1.3533	243.7	265.3	0.34	0.34
MiSeq_1	0.87	1.7521	14.2	5.5	4.58	3.57	0.7311	62.9	19.5	9.06	5.24	0.1306	2.8	0.6	0.60	0.30	0.6482	147.9	159.9	0.34	0.34
SRR554369_1	0.17	1.8671	3.6	1.8	2.05	1.50	1.0221	41.2	13.5	2.13	1.05	0.2389	6.5	0.8	1.58	0.92	0.9131	28.6	31.1	0.31	0.31

↵

Table 3: Compression, time and memory usage in the order preserving regime (Genozip and Jarvis 3) and the non-order preserving regime (Genie) on SE datasets.

Compression ratios are in bits per base (bpb). “ctime” (resp. “dtime”) stands for compression (resp. decompression) time, and is expressed in seconds. “cmem” (resp. “dmem”) stands for peak compression (resp. decompression) memory usage and is expressed in gigabytes (=10⁹ bytes).

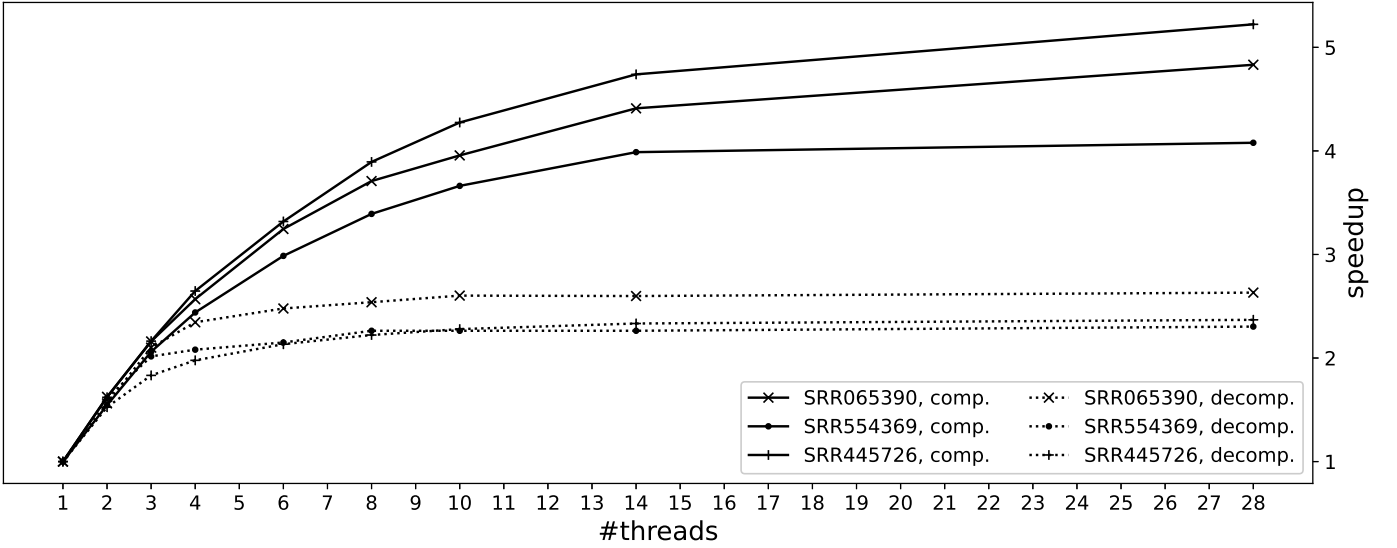

Figure 1: Relative compression and decompression times in the function of the number of threads, with respect to single-threaded execution.

Three datasets were used: SRR554369_PE_ORD, SRR065390_PE_ORD, SRR445726_SE (i.e., SRR554369 and SRR065390 were compressed in the order preserving PE mode, and SRR445726 was compressed in the order non-preserving SE mode).

3 Additional tables and figures

Table 1 (resp. 2) shows speed, ratio, and memory usage of PgRC and its competitors for all SE and PE datasets in order non-preserving (resp. preserving) mode. Mstcom excels in compression ratio, whereas SPRING’s strong point is its low memory requirement when decompressing large datasets, which might be critical in case of low-end workstations. PgRC 2.0 stands out for its decompression speed. The slow compression of PgRC 1.1 and Mstcom calls into question their practicality. Additionally, Mstcom shows excessive demand for memory and is the slowest in decompression. The choice between SPRING and PgRC 2.0 is not so clear. In decompression, the first offers better memory utilization while the latter provides higher speed. A better compression ratio might be a factor in favor of PgRC 2.0.

Extra results from a few recent (and not tested in the main paper) competitors, Genozip in two modes, Genie and Jarvis 3, are presented in Table 3. It should be first noticed that Genie cannot preserve the read order, hence its results are not directly comparable with the others in this table. Having said that, we admit that Genie’s results are quite competitive in many cases, with compression ratio by 26% worse on average than PgRC2 in the SE mode (and winning on two datasets), while its compression (resp. decompression) times are worse than PgRC2’s ones by 33% and 53%, respectively (all presented averages are geometric means). Genozip and Jarvis 3 cannot compress the DNA stream alone, but the actual fractions of the compressed stream in the archive are presented, so we used the DNA sizes for the comparison. Unfortunately, for the same reason, their timings are not directly comparable to PgRC2. With such a proviso we can conclude that Genozip’s compression times are rather competitive, but decompression times are less attractive, and its compression ratios in the default (resp. best) mode in 15 (resp. 10) out of 16 cases were more than twice worse than PgRC2’s. Jarvis 3 is often at least 3 times slower than PgRC2 (SE_ORD) in compression, and sometimes the gap reaches an order of magnitude. In decompression, it is even less competitive as its compression model is symmetric.

As a final note, we point out that the applicability of these three tools extends beyond just FASTQ compression. Genozip is the most versatile one, handling multiple bioinformatics formats, including FASTA, FASTQ, BAM, SAM, CRAM, VCF, GFF, and more. Genie is able to transcode from FASTQ and SAM to MPEG-G records and vice versa, and Jarvis 3 can compress FASTA and FASTQ.

Fig. 1 shows the compression and decompression speedup with varying the number of threads from 1 to 28. The speed of decompression does not improve much beyond 6 threads (but it might with more efficient disk I/O). In compression, the gain from multi-threaded implementation plateaus out quite quickly. The speedup does not reach the factor of 5 (times) for 14 threads in the case of the tested datasets. Such behavior can be mostly explained by a weaker parallelization of selected phases of the algorithm.

Fig. 2 presents the fraction of total PgRC’s processing time per phase, using 1 and 28 threads, respectively. The total

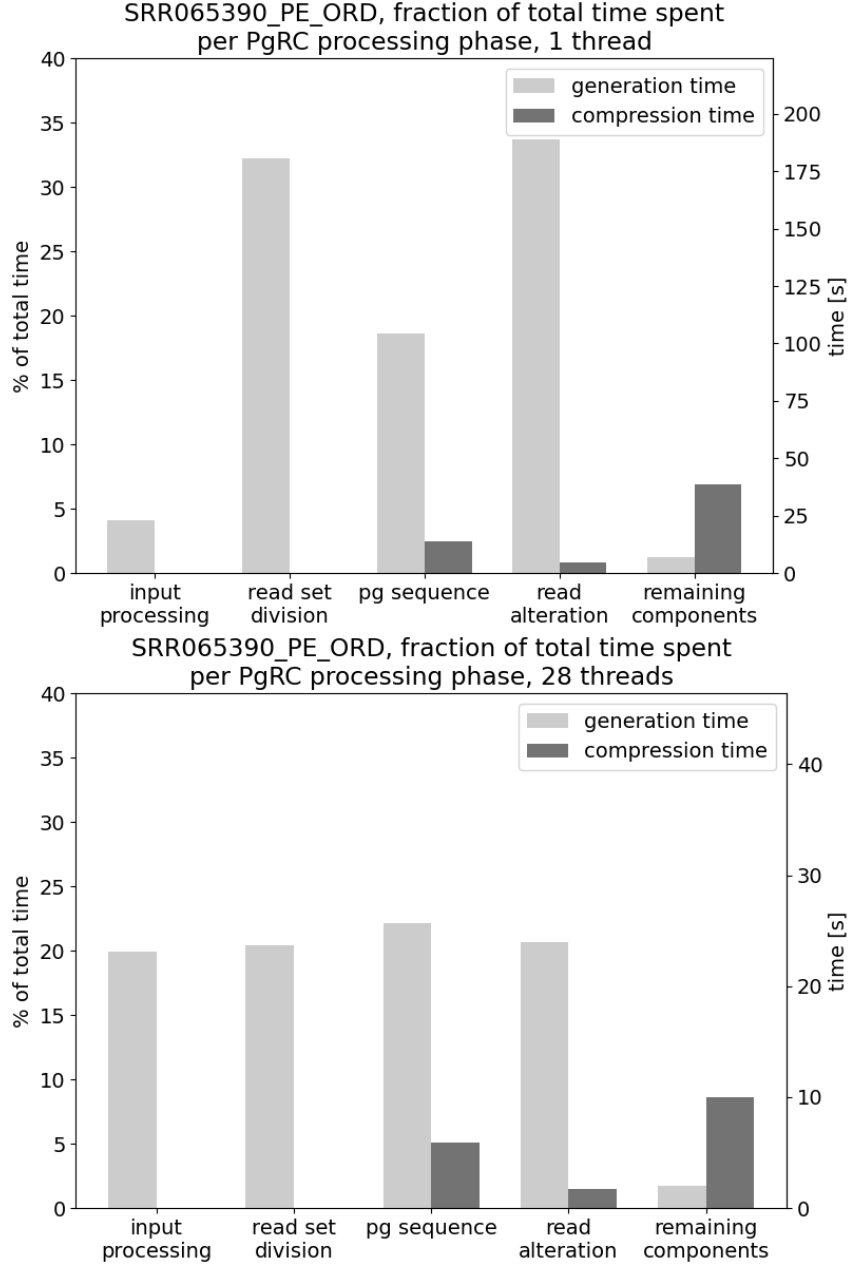

Figure 2: Fraction of the total time of the PgRC 2.0 compression process per phase, for SRR065390 compressed in the PE_ORD mode. The top (resp. bottom) figure presents results for 1 thread (resp. 28 threads). The left bar of each pair concerns producing “raw” components, and the corresponding right one compressing it with a backend compressor. The phase “pg sequence”, the compression bar, includes also the var-length encoding, which takes about 0.5s. The “read alteration” term comprises operations producing and compacting alignment data: RC vs direct alignment flags, mismatch counts per read, mismatch symbols and mismatch positions within a read. “Remaining components” refer to raw read positions (ordered) and read pairing data.

compression time for the presented dataset, SRR065390, in the PE_ORD mode, is 560.5s (resp. 116.0s) for 1 thread (resp. 28 threads). Using 28 threads gives thus a speedup by a factor of 4.8 on this dataset. We point out that the relative impact of reading the input data (from an SSD) grew significantly: close to 5 times. The read set division is over 7.6 times faster with 28 threads, which translates to a reduction from 32.2% to 20.4% for the fraction of the total time. Building the pseudogenomes using 28 threads is 4 times faster, but the LZMA compression on them only 2.3 times. However, the pg compression time for PgRC 2.0 even with 1 thread is over 15 times shorter than the corresponding time for PgRC 1.1. This spectacular improvement was mainly possible due to the variable-length encoding (VLE) phase, which is almost free

dataset	pg seq.	VLE +			VLE +		total	
	raw size	LZMA	LZMA	ratio	LZMA	LZMA	time	time
	[Mb]	ratio	ratio	gain	time	time	gain	gain
ERR532393_1_SE	451.439	0.224	0.220	0.989	18818	466332	24.781	5.560
MiSeq_1_SE	10.046	0.200	0.202	1.002	237	3850	16.245	1.319
SRR065390_1_SE	132.443	0.222	0.226	1.006	4202	103460	24.622	2.491
SRR1294116_SE	293.395	0.236	0.239	1.008	10032	282585	28.168	3.815
SRR445718_SE	210.484	0.203	0.205	1.002	10242	170264	16.624	3.026
SRR445724_SE	406.699	0.193	0.192	0.999	26360	356076	13.508	3.171
SRR445726_SE	353.869	0.192	0.192	1.000	21766	302309	13.889	3.050
SRR490961_SE	164.918	0.196	0.199	1.004	8205	122437	14.922	2.260
SRR490976_SE	258.965	0.180	0.183	1.004	16109	199259	12.369	2.992
SRR554369_1_SE	10.826	0.230	0.233	1.008	251	4549	18.124	2.438
SRR635193_1_SE	107.114	0.237	0.242	1.012	2729	84178	30.846	3.530
SRR689233_1_SE	52.599	0.226	0.228	1.003	1277	35096	27.483	2.207
SRR870667_1_SE	2994.435	0.139	0.124	0.943	233018	3798911	16.303	6.237
ERR532393_PE	708.309	0.224	0.218	0.990	31203	786921	25.219	4.967
MiSeq_PE	19.208	0.200	0.203	1.002	452	9014	19.942	1.324
SRR689233_PE	77.381	0.225	0.225	1.000	1963	55439	28.242	1.925
SRR635193_PE	164.699	0.235	0.240	1.005	5218	139321	26.700	2.916
SRR065390_PE	163.645	0.224	0.229	1.004	5973	132747	22.225	2.137
SRR554369_PE	13.302	0.226	0.230	1.006	295	5788	19.620	2.213

Table 4: The impact of the variable-length encoding (VLE) of the pseudogenome before LZMA compression (which uses 2 threads). The pseudogenome, built over high-quality reads, is already made somewhat denser, as long enough RC-matches belonging to it are already found and removed. The “ratio gain” column results below 1 mean that the VLE preprocessing hampers the compression ratio of this data stream, but only in one case the loss is quite significant (SRR870667_1_SE). The “time gain” and “total time gain” columns, on the other hand, witness the speedup achieved thanks to VLE.

in the processing time, and not only shortens the input for the LZMA, but also speeds up match searching over it, thanks to its more “dense” representation. An additional reason is that the pseudogenomes in v2.0 are smaller due to parameter tuning (M), allowing for aligning reads with a larger amount of mismatches than before. To give another perspective, we note that in v1.1 the time fraction for this stage, i.e., “pg sequence, compression time”, was 23.5%; while in v2.0 it drops to 2.5% (1 thread) and 5.1% (28 threads), respectively. Finally, preparing the read alteration data is by a factor of 7.5 faster with 28 threads (while the compression time reduced 2.5 times). The relative impact of this stage, with regard to the total compression time, dropped from 34.4% (1 thread) to 25.7% (28 threads).

To have a more comprehensive overview of the impact of VLE preprocessing before LZMA compression over the pseudogenome, as well as threading for particular phases of the compressor, see Tables 4 and 5, respectively. For example, we can notice in Table 5 that the alignment phase is the easiest to parallelize, with the speedup factor usually over 15, for the pg construction it drops to around 7 and is lowest for the streams compression phase, being around 2-fold. It should also be pointed out that some stages of the whole compressor (e.g., the I/O operations) are not parallelized and their timings are not presented in this table.

Let us now discuss briefly our concurrent hash table (HT), used for finding matches over the pseudogenome. Our HT data structure allows for thread interference and spurious false insertions, but all its entries which store values (i.e., seed positions in the pseudogenome) going beyond the pseudogenome boundaries are zeroed for safety, once the HT is built. For this reason, the only possible danger is missing too many matches (that would hamper the overall compression performance). The fraction of lost matches in the multi-threaded implementation, run with 28 threads, as compared to a single-threaded one, is up to 0.095% (SRR445726_SE) over our test collection. On the other hand, the fraction of lost insertions is even less, up to 0.0017% (SRR445718_SE). Interestingly, those lost matches actually translated to a slight compression *gain*, on the order of 0.1% (which suggests that the rejected matches were of rather low quality and the corresponding reads tended to be better compressed in other stages of the compressor). Overall, we can conclude that in our application an unsafe multi-threaded hash table may be competitive against a correct, multi-threaded one (as the alignment phase, mentioned above, is sped up by a factor from 12 to 19, which couldn’t be possible without a highly

dataset	t1→t28	pg	pg	pg	match	match	match	comp	comp	comp
	total time	time	time	time	time	time	time	time	time	time
	gain	(t28)	(t1)	gain	(t28)	(t1)	gain	(t28)	(t1)	gain
ERR532393.1.SE	5.307	25256	213029	8.435	11801	214745	18.197	20711	49758	2.402
MiSeq_1.SE	5.640	5277	42932	8.136	804	15100	18.781	434	1027	2.366
SRR065390.1.SE	4.422	26425	165524	6.264	5320	83305	15.659	5846	14734	2.520
SRR1294116.SE	5.079	35028	273344	7.804	8812	144241	16.369	12207	31089	2.547
SRR445718.SE	5.263	25018	178564	7.137	9102	176279	19.367	11836	26071	2.203
SRR445724.SE	4.956	48983	329625	6.729	15993	304663	19.050	29945	58538	1.955
SRR445726.SE	5.143	43541	294875	6.772	15828	306177	19.344	24893	49478	1.988
SRR490961.SE	5.204	28463	211166	7.419	10241	193984	18.942	10115	22657	2.240
SRR490976.SE	4.494	28819	159548	5.536	9677	182637	18.873	18210	33742	1.853
SRR554369.1.SE	3.298	1190	5544	4.659	190	2343	12.332	383	674	1.760
SRR635193.1.SE	4.173	12084	88836	7.352	1250	19998	15.998	3623	9606	2.651
SRR689233.1.SE	5.381	11085	89151	8.042	2447	43617	17.825	1944	5023	2.584
SRR870667.1.SE	5.004	253713	1973566	7.779	42247	825209	19.533	244655	463415	1.894
SRR689233.PE	5.190	21758	170815	7.851	5020	85350	17.002	6017	17648	2.933
SRR635193.PE	3.950	20941	165802	7.918	2456	37888	15.427	13870	38789	2.797
SRR554369.PE	4.135	1448	11032	7.619	300	4311	14.370	571	1057	1.851
SRR065390.PE	4.761	38000	271935	7.156	10316	175135	16.977	9886	26633	2.694
MiSeq_PE	6.462	11624	98277	8.455	2990	57598	19.264	1390	3914	2.816
ERR532393.PE	4.945	46778	374599	8.008	21310	381741	17.914	40660	98088	2.412

Table 5: The speedup from multithreading. The second leftmost column shows the gain for the whole compression process, when the number of threads grows from 1 to 28. Likewise, the further columns with “t1” (resp. “t28”) present timings for 1 thread (resp. 28 threads). “pg time” is the pseudogenome construction time, “match time” is the time of aligning reads onto the (high-quality reads) pseudogenome, and “comp time” stands for the compression time for all the resulting streams, using backend compressors.

concurrent hash table implementation).

One of the novelties in PgRC 2.0 is also quality score based filtering, which discriminates between two classes and classifies each read as either a high-quality candidate or a low-quality one (the candidates will yet be divided into genuine high-quality reads and low-quality ones, in the “read set division” phase). The idea is to have a possibly fast crude filter, which already eliminates a significant fraction of reads. Despite the fact that the fraction of the reads which are considered in this stage of low quality is relatively low (21% as the median fraction and 18.1% on average, which contrasts with 0.5% and 4.7% in PgRC 1.1 based on N-containing reads elimination), the speedup of the subsequent phase is significant. More precisely, the filter allowed to speed up the read set division phase by an average (resp. median) factor of 1.81 (resp. 1.55) and shrink the total time by an average (resp. median) factor of 1.11 (resp. 1.08); the averages are taken over a collection of 19 datasets. On the other hand, the impact of this idea on the compression ratio is less beneficial; the ratio gets worse by 0.3% on average (the median by 0.02%), but it varied from dataset to dataset. For two PE and three SE datasets there was no difference and for two SE datasets the loss in ratio was around 2.5%.

4 What is new in PgRC 2.0

Most changes listed in this section were discussed in the main paper and in the previous section of Supplementary Material, but we present them here together, to give an overall overview of the progress in development of our software.

The most important changes introduced in PgRC after version 1.1 are as follows.

- Most stages of compression and decompression are now parallel (obtained with aid of the OpenMP library).
- Implemented variable-length DNA encoding – such DNA preprocessing technique allows finding LZ-matches much faster.

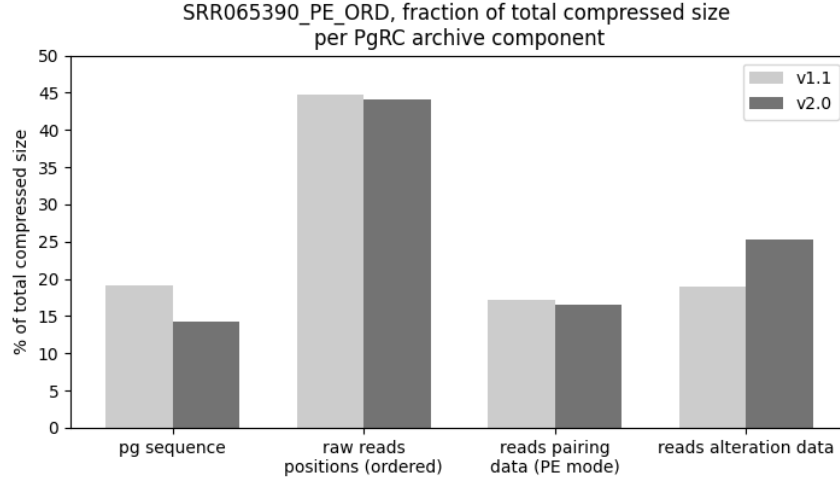

Figure 3: Fraction of the total size of PgRC’s archive per component. The figure presents the results for SRR065390_PE_ORD (i.e., SRR065390 was compressed in the order preserving PE mode).

- Obtained a speedup in the phase of establishing a set of high-quality reads (called briefly “reads set division” in Fig. 2) – more than $1.5\times$ for most datasets – through applying a simplified quality stream based reads filtering (see the section “Materials and Methods” in the main paper for details).

Minor changes:

- Improved encoding of mismatches in aligned reads:
  - ★ Number of mismatches encoded by backend compressors now uses two streams (separate zero flags stream).
  - ★ Encoding streams of mismatches offsets selecting the best backend compressor between: ppm, FSE and range coder (which is configured to encode the first offsets separately).
  - ★ The alphabet is reordered for exclusive encoding of mismatches to consider frequency of mismatch symbols.
- Obtained a speedup in overall compression (by $>10\%$ in SE modes and $>4\%$ in PE modes) via decreasing the M parameter (from 5 to 3) to allow more mismatches in aligned reads. The effect of the change is distinctly visible in Fig. 3. The plot shows fractions of the whole archive taken by particular components in PgRC 2.0 and 1.1. The allowed number of mismatches translates into a trade-off between the size of the final pg sequence and the size of the data about mismatches in aligned reads. In the new PgRC, compression ratio remains high due to improved encoding of mismatches, and compression time is reduced since squeezing the pg sequence is generally slower (cf. Fig. 2).
- For most streams, tuning selection and internal parameters of backend compressors:
  - ★ PPM and LZMA (from <https://7-zip.org/sdk.html>),
  - ★ range coder (implemented as a part of fqzcomp v4.6 compressor <https://github.com/jkbonfield/fqzcomp>),
  - ★ FSE (<https://github.com/Cyan4973/FiniteStateEntropy>).
